# Personal

**Published:** 1918-11

**Authors:** 


### PERSONAL

Announcement of removal, travel, and other matters of interest are requested. Please report errors in the listing of any physician in the State and other directories, that we may co-operate with the State Society in securing a correct list.

---

Dr. Grover Wende spent the month of October in Washington on government work.

Dr. Lucien Howe was elected President of the American Opthamalogical Society at its annual meeting.

A subscription dinner was given Dr. Wm. D. Johnson at the Saturn Club on October 2d by the members of the Buffalo Academy of Medicine.

Dr. William H. Mansperger of Buffalo has returned from a motor trip through the Adirondacks.

Dr. Lillian Craig Randall of New York City, formerly of Buffalo announces the removal of her office to 570 West 156th St.

Dr. George E. Blackham of Dunkirk had his left shoulder broken in an automobile collision. He was taken to the Brooks Memorial Hospital.

